# Infections Caused by HRSV A ON1 Are Predominant among Hospitalized Infants with Bronchiolitis in São Paulo City

**DOI:** 10.1155/2017/3459785

**Published:** 2017-05-24

**Authors:** Sandra E. Vieira, Luciano M. Thomazelli, Milena de Paulis, Angela E. Ferronato, Daniele B. Oliveira, Marina Baquerizo Martinez, Edison L. Durigon

**Affiliations:** ^1^Faculdade de Medicina da Universidade de São Paulo, São Paulo, SP, Brazil; ^2^Instituto de Ciências Biomédicas da Universidade de São Paulo, São Paulo, SP, Brazil; ^3^Hospital Universitário da Universidade de São Paulo, São Paulo, SP, Brazil; ^4^Faculdade de Ciências Farmacêuticas da Universidade de São Paulo, São Paulo, SP, Brazil

## Abstract

Human respiratory syncytial virus is the main cause of respiratory infections in infants. Several HRSV genotypes have been described.* Goals*. To describe the main genotypes that caused infections in São Paulo (2013–2015) and to analyze their clinical/epidemiological features.* Methods*. 94 infants (0–6 months) with bronchiolitis were studied. Clinical/epidemiological information was collected; a search for 16 viruses in nasopharyngeal secretion (PCR-real-time and conventional, sequencing, and phylogenetic analyses) was performed.* Results*. The mean age was 2.4 m; 48% were male. The mean length of hospital stay was 4.4 d (14% in the Intensive Care Unit). The positive rate of respiratory virus was 98.9%; 73 cases (77.6%) were HRSV (76,7% HRSVA). HRSVA formed three clusters: ON1 (*n* = 34), NA1 (*n* = 1), and NA2 (*n* = 4). All HRSVB were found to cluster in the BA genotype (BA9-*n* = 10; BA10-*n* = 3). Clinical analyses showed no significant differences between the genotype AON1 and other genotypes.* Conclusion*. This study showed a high rate of HRSV detection in bronchiolitis. HRSVA ON1, which has recently been described in other countries and has not been identified in previous studies in the southeast region of Brazil, was predominant. The clinical characteristics of the infants that were infected with AON1 were similar to infants with infections by other genotypes.

## 1. Introduction

HRSV infections are frequent worldwide, and the most severe cases mainly affect children during the first year of life, elderly and immunocompromised individuals. HRSV is the most common causal agent of respiratory infections in infants, which occur at predictable annual seasons [1, 2].

HRSV is an enveloped virus (genus* Orthopneumovirus*, family Pneumoviridae) [3]. Its genome consists of a nonsegmented single-stranded RNA that encodes 11 proteins. The surface proteins F and G are important antigenic targets of neutralizing antibodies. Variations in the G protein and in the gene regions that encode this protein allowed for the classification of HRSV into two subgroups (A and B) and into many genotypes. Different HRSV genotypes of the two subgroups generally cocirculate during a season in the same region, and the predominant genotypes are replaced by others in subsequent years [4, 5].

Such antigenic and genetic variability allows the virus to escape immunity acquired by the population of patients that have been subjected to previous infections. Several genotypes have been described in subgroups A (GA1 to GA7, SAA1 and NA1, NA2) and B (URU1, URU2, and GB1 to GB4, SAB1 to SAB3, and BA1 to BA13). The recently described genotype HRSV A ON1 is characterized by the duplication of 72 nucleotides in gene G. After being reported in Canada, HRSVA ON1 has also been identified in Europe, India, Africa, South America, and Asia [6–11].

Knowledge on the molecular epidemiology of HRSV infections is important to assess the clinical implications of infections by different genotypes. The clinical presentation, severity, response to treatment, and prophylaxis are among these implications. The occurrence of genotypes that have not been previously identified leads to concerns about the severity of new cases or an increased number of infected individuals when considering the possible absence of immunological memory in the affected population.

In this study, the authors describe the main genotypes of HRSV A and B, which caused infections in infants hospitalized in the University Hospital of Universidade de São Paulo in São Paulo city, from 2013 to 2015. The genotype characteristics and clinical and epidemiological features of HRSV are analyzed, and the infections caused by the new genotype HRSV AON1 are compared to other genotypes that were circulating during the study period. To our knowledge, this is the first report to perform an analysis of the association between clinical features and genotypes in infections caused by HRSV A ON1 in the southeast region of Brazil.

## 2. Methods

This study included 124 infants aged between zero and six months with diagnoses of bronchiolitis that were admitted to the Pediatric Clinic Division of the University Hospital of the University of São Paulo between 2013 and 2015. The infants were enrolled in the study by one of the authors (MP) who collected nasopharyngeal secretions after written consent was obtained from the child's parents. On examination, the clinical and socioeconomic backgrounds, clinical signs/symptoms, and diagnosis at admission were recorded on a standard form. Diagnosis of bronchiolitis was defined as the first wheezing crisis, beginning no more than 3 days before hospital admission.

For clinical analysis, infants diagnosed with or suspected of having bacterial or fungal infections and those who received antibiotics, macrolides, or antifungals prior to or during hospitalization were excluded. Infants with codetection of respiratory viruses were also excluded from the clinical analysis.

The project was approved by the Research Ethics Committee of the University Hospital of the University of São Paulo (1011/10) and was funded by Fundação de Amparo a Pesquisa do Estado de São Paulo (12/22854-9).

Nasal wash fluid was obtained after washing the nostrils with 3 ml of a saline solution and collecting the suctioned specimen in a cup within a maximum of 24 hours after admission. The samples were shipped within 48 hours under refrigeration and analyzed at the Laboratory of Clinical and Molecular Virology of Institute of Biomedical Sciences, University of São Paulo, by in-house singleplex real-time RT-PCR assays for the detection of 16 common respiratory viruses (HRSV A and B; human rhinovirus, enterovirus, human metapneumovirus, parainfluenza virus 1, 2, 3 and 4; adenovirus, influenza virus A and B, bocavirus, and coronavirus OC43, NL63, HKU1, and 229E). Viral RNA was extracted with an automatic NucliSens easyMAG extractor (Biomerieux, Inc., Durham, NC, USA) according to the manufacturer's instructions. The one-step qPCR was carried out with the AgPath-ID one-step RT-PCR kit in the 7300 Real-Time PCR System (Applied Biosystems™, Foster City, CA, USA) with primers previously described by Sakthivel et al., 2012.

HRSV-positive samples were amplified by traditional PCR in two steps for DNA sequencing: cDNA was synthesized using the Super Script III kit (Applied Biosystems, Foster City, CA, USA) according to the manufacturer's instructions. The second hypervariable region of the G protein gene PCR was carried out with the primers Gr5_fwd (5′-CTGGCAATGATAATCTCAACTTC-3′) and FV_rev (5′-GTTATGACACTGGTATACCAACC-3′) in a 10 *μ*L mixture that contained 5 *μ*L of 10x PCR buffer, 25 mM of each dNTP, 25 pmol of each primer, and 1.5 U of Platinum Taq DNA Polymerase (Invitrogen, Carlsbad, CA, USA) for a final volume of 50 *μ*L. The amplification was performed in a GeneAmp PCR System 9700 thermocycler (Applied Biosystems, Foster City, CA, USA). A second step with Seminested PCR was carried out using the F1AB_rev (5′- CAACTCCATTGTTATTTGCC-3′) primer corresponding to bases 3–22 of the F gene. The traditional PCR assays were performed with the following program: 95°C for 5 minutes, followed by 35 cycles, each composed of 30 sec at 95°C, 30 sec at 55°C, and 45 sec at 72°C, and finally 7 minutes of extension at 72°C. The amplified products were analyzed by agarose gel electrophoresis and visualized under UV light after staining with ethidium bromide. The amplified products of gene G were ≈490 bp, were purified by ExoSap-IT (Affymetrix, Inc., USA), and were submitted to a cycle sequencing reaction (Sanger) using the Bigdye terminator kit (Applied Biosystems, Foster City, CA, USA) and GR5, FV or F1AB primers in a 3100 DNA Sequencer (Applied Biosystems, Foster City, CA, USA). Both strands of each amplicon were sequenced at least twice. Sequence editing, alignments, and phylogenetic analyses were performed with MegAlign 5.03 v software (DNAStar, Inc., Madison, Wisconsin, USA). Standard published sequences from subgroups A (accession numbers from KY828387 to KY828428) and B (accession numbers from KY828374 to KY828386) were downloaded from GenBank as references of different lineages and genotypes.

### 2.1. Statistical Analysis

The results of the analyses of the clinical and demographic characteristics as well as the categorical variables are presented as absolute numbers and percentages, and the continuous variables are presented as the means and standard deviations. For studies of the associations between the categorical variables, the chi-square test, and Fisher's exact test were used. For associations between the continuous variables, Student's *t*-test was used. The null hypothesis was rejected when the probability was less than 5% (*P* < 0.05). We used IBM SPSS Statistic software, version 23.

## 3. Results

Of the 124 infants selected, 94 were studied after the exclusion of 30 cases (infants with clinical diagnoses or suspicion of infection by bacteria or other agents as well as those who received antibiotics).

**Figure 1:**
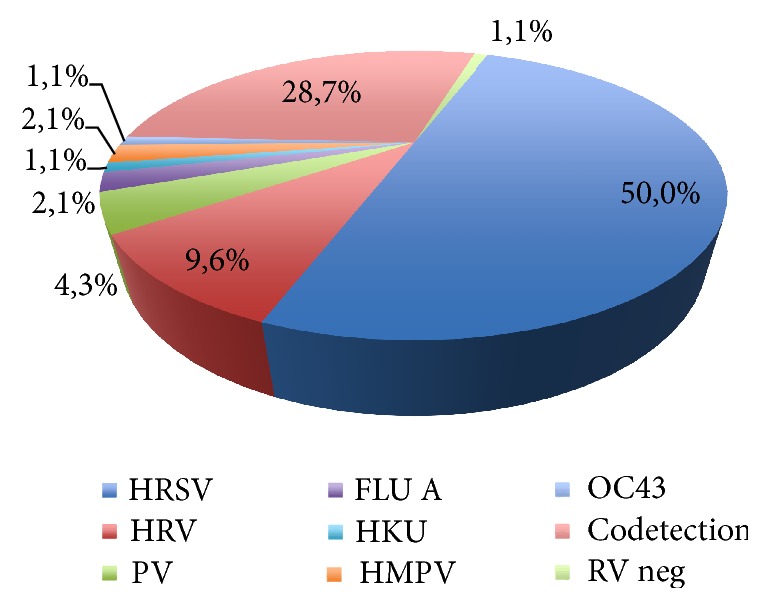
Etiological analysis of the 94 cases of bronchiolitis in infants younger than 6 months. HRSV = human respiratory syncytial virus; HRV = human rhinovirus; PV = parainfluenza virus; Flu A = influenza virus A; HKU = coronavirus HKU; HMPV = human metapneumovirus; OC43 = coronavirus OC43; RV neg = result was negative for respiratory viruses.

**Figure 2:**
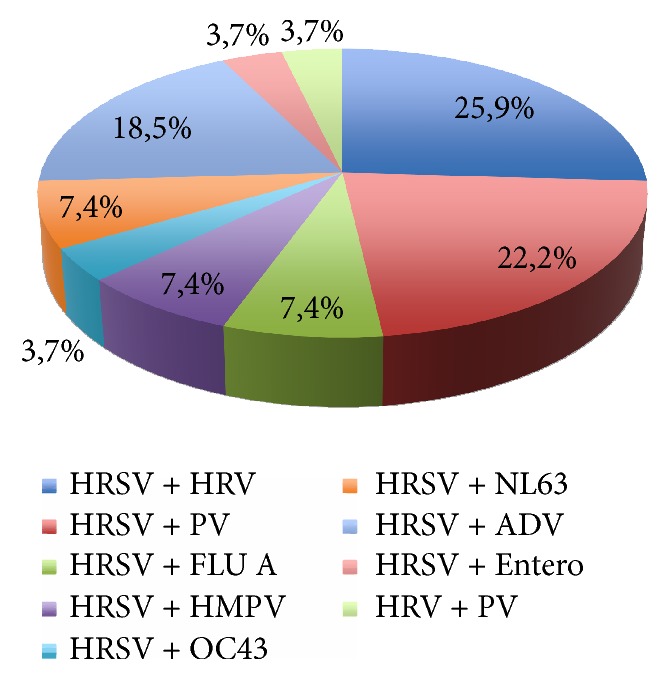
Etiological analysis of 27 cases with respiratory virus codetection. HRSV = human respiratory syncytial virus; HRV = human rhinovirus; PV = parainfluenza virus; Flu A = influenza virus A; HKU = coronavirus HKU; HMPV = human metapneumovirus; OC43 = coronavirus OC43; NL63 = coronavirus NL63; ADV = adenovirus; Entero = enterovirus.

The mean age of the infants was 2.4 months (SD = 1.6) and 48% were male. The mean length of hospital stay was 4.4 days (SD = 3.6) and 14% were admitted to the Intensive Care Unit. The positivity of respiratory virus detection was 98.9%; 73 (77.6%) samples were positive for HRSV, 47 were single-agent, and 26 showed codetection with other respiratory viruses. Cocirculation of HRSV A and B was observed, with a predominance of HRSVA (76.7%). The results of the viral agent searches in the studied 94 cases of HRSV are shown in Figures 1 and 2.

**Figure 3:**
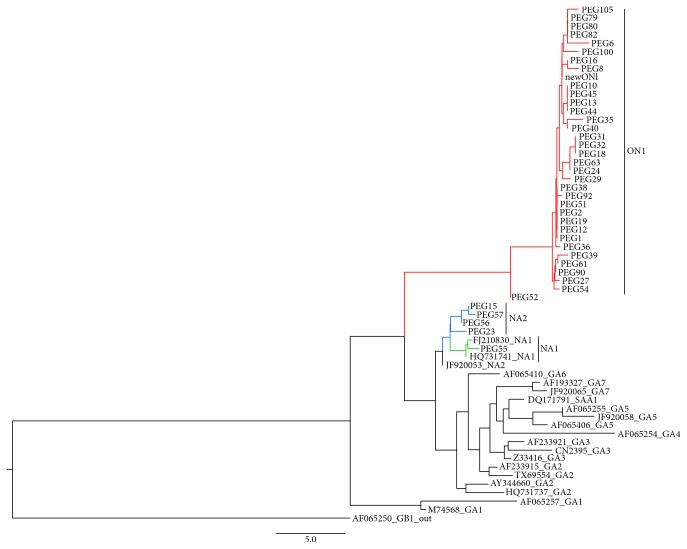
Genotype tree: the topology of the HRSV A tree shows the study samples identified (PEG) compared to the specimens from GeneBank, identified by their access number. Clades in red show samples that belong to genotype ON1. Clades in blue show samples that belong to genotype NA2, and clades in green show samples that belong to genotype NA1.

**Figure 4:**
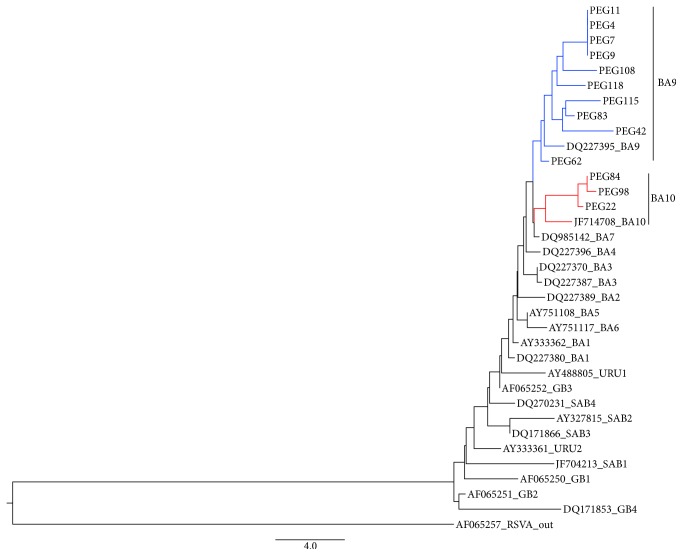
Genotype tree: the topology of the HRSV B tree shows the study samples identified (PEG) compared to the specimens from GeneBank, identified by their access number. Clades in red show samples that belong to genotype BA10. Clades in blue show samples that belong to genotype BA9.

Fifty-two samples of HRSV were sequenced (34 A ON1, 10 BA9, 4 NA2, 3 BA10, and 1 NA1). Genotype trees are presented in Figures 3 and 4. The HRSV A isolates formed three clusters (NA1, NA2, and ON1 genotypes); most of the HRSVA isolates were found to cluster in the ON1 genotype. The ON1 cluster included 40 isolates, the NA1 cluster included 2 isolates, and the NA2 cluster included 4 isolates. They showed a 0.004–0.113 sequence* p*-distance at the nucleotide level, but there was a 0.000–0.175* p*-distance at the amino acid level compared to the ON1 prototype strain (JN257694). The sequenced HRSVB isolates were found to cluster in the BA genotype. The HRSV B isolates formed two subclusters, identified as the BA-9 and BA-10 genotypes. The BA-9 cluster included 10 isolates, and the BA-10 cluster included 3 isolates. They showed a 0.026–0.052 sequence* p*-distance at the nucleotide level, but there was a 0.045–0.084* p*-distance at the amino acid level compared to the BA prototype strain (AY333362).

The comparative clinical analyses included 32 infants with a HRSV single infection (22 AON1 and 10 other genotypes) and showed no significant differences between these subgroups (Table 1).

## 4. Discussion

The present study showed the strong predominance of HRSV infections in infants hospitalized with bronchiolitis, predominance of the HRSVA ON1 genotype, and occurrence of the NA1 and NA2 genotypes, previously unidentified in southeast region of Brazil.

The high occurrence of HRSV among infants was expected due to the inclusion criteria that selected children under 6 months old and the exclusion of those who had diagnoses or suspicion of infection by nonviral agents. In fact, HRSV is recognized as the most frequent etiologic agent in bronchiolitis during the first year of life [1].

The use of molecular methods for conducting viral research in the selected cases showed low occurrences of 6 other respiratory viruses and a high prevalence of respiratory virus codetections, most of which involved HRSV, which was the main viral agent in both single infections and in codetection.

Cocirculation of HRSV A and B was observed, with a predominance of HRSVA (76.7%), as reported in most studies conducted in other countries [5, 7, 12]. Previous studies performed by this group of researchers, in the same service, showed the cocirculation of different genotypes of HRSV A and B during the same viral season. An analysis from 1995 to 2006 showed the predominance of group B only in 1999 in the State of São Paulo [13].

After genotype HRSV A ON1, BA9 was the second most frequent genotype identified in the present study. The genotypes that predominated in previous seasons were not detected, such as GB1, GB3, GA2, and GA5. In a previous study, the authors included HRSV samples that were collected from hospitalized children in the State of São Paulo until seven years before this study. They showed important nucleotide substitutions in the GA2 genotype that are genetically close to the NA1 and NA2 genotypes identified here between 2013 and 2015 [14].

Since the first year of the study (2013), the genotype HRSVA ON1 was predominant, but the genotypes NA1 and NA2 and genotypes BA9 and BA10 also circulated. In subsequent years (2014 and 2015), the genotype ON1 remained predominant and a unique representative of HRSV A, but there was cocirculation with the BA9 and BA10 genotypes, with predominance of the BA9 genotype among HRSV B.

In molecular analysis, most of the sequenced HRSVA isolates were found to cluster in the ON1 genotype with prototype reference strain JN257694, which was first reported in Ontario, Canada [6]. The sequenced isolates had the signature 72-bp duplication in the G protein when aligned with representative sequences from all of the A subgroup (GA1 to GA7, NA1, NA2, and ON1) from GenBank. The Brazilian HRSV A isolates were most closely related to isolates from the United States, Kenya, and New Zealand.

All of the sequenced HRSVB isolates were found to cluster in the BA genotype, with prototype BA reference strain AY333362, which was first reported in Buenos Aires, Argentina [15]. The sequenced isolates had the signature 60-bp duplication in the G protein when aligned with representative sequences from all of the B subgroup (GB1 to GB4, SAB1 to SAB4, URU1 and URU2, and BA-1 to BA-13) from GenBank. The Brazilian HRSV B isolates were most closely related to isolates from the United States, New Zealand, and Vietnam.

Some authors suggest the occurrence of a greater number of cases during seasons in which new genotypes predominate. This could be a result of the absence of immunity acquired by the population against the new genotypes [4, 15]. Genotype A ON1 shows a duplication of 72 nucleotides in the C-terminal third of the G gene. The region of the G protein encoded by this gene sequence is targeted by specific genotype neutralizing antibodies, which may contribute to the escape of the virus from the population immunity induced by previous contact with other genotypes. However, according to the National Registry of Hospitalization Cases, there was no increase in the frequency of hospitalizations of infants diagnosed with bronchiolitis between 2013 and 2015 in Brazil and the State of São Paulo compared to the previous five years [16]. It is possible that, acting as a controller, the immunity of the population only contributes to the selection of genotypes that replace each other without necessarily increasing the number of cases when genotypes without recent incidences appear.

The demographic and clinical characteristics of the infants that were infected with genotype AON1 were similar to the characteristics of the other genotypes. Exclusion of infants with viral codetection and those who received antimicrobials during hospitalization made it possible to avoid confounding factors in the comparative analysis of the severity between the genotypes.

A mean age at 2 months showed the precocity of the infection, which was independent of the infecting genotype. Clinical characteristics that could differentiate the initial presentation, such as the presence of cough, fever, dyspnea, and apnea crises, also showed similar prevalence among the genotypes. Some prognostic factors that are relevant in respiratory infections in infants were also similar, such as exposure to tobacco smoke and breastfeeding. Severity, as analyzed by the hospitalization time, need and duration of oxygen therapy and mechanical ventilation, and hospitalization in the Intensive Care Unit, was also similar regardless of the infecting genotype.

Despite the limited number of cases, the results suggest that the HRSV A ON1 genotype was not associated with specific clinical characteristics and was therefore clinically indistinguishable from the other genotypes. These results need to be confirmed by more extensive analyses but are consistent with a previous German study that found no clinical differences between infections by other HRSV genotypes [17]. Although nucleotide variation in this region is important for viral antigenicity, other regions may be more relevant determinants of infection severity. In addition, other factors inherent to the host and the environment must be considered [18].

On the other hand, an epidemiological study carried out in Vietnam compared community-acquired infections and nosocomially acquired infections and showed a greater severity of the respiratory condition in children infected with HRSV ON1 compared to those infected with NA1 with consideration of the clinical severity and occurrence of pneumonia. In that study, all children used antibiotics, which may have created a bias since nonexcluded bacterial coinfections could impact clinical evolution. Additionally, the age group was a differential factor since children up to 5 years of age were included, including infants with bronchiolitis and also cases of posterior HRSV infections [19]. Other authors found fewer signs of severity in children infected by HRSV A ON1 compared to other HRSV genotypes, such as NA1 [7], GA2, and BA [20].

Another important aspect is the need for clinical follow-up studies that can assess the possible impacts of infections by different genotypes on the development of recurrent wheezing and asthma in the years following infections.

## 5. Conclusion

The present study showed a high rate of HRSV detection in infants hospitalized with bronchiolitis. Five genotypes were found, with a predominance of genotype A ON1, which was recently described in other countries and not identified in previous studies in the southeast region of Brazil. The clinical and epidemiological characteristics of infants that were infected with HRSV A ON1 were similar to infants with infections by other genotypes identified in the study.

## Figures and Tables

**Table 1:** Comparative analysis between the demographic and clinical characteristics of infants with HRSV infection caused by ON1 and by other genotypes.

	All genotypes *N* = 32	HRSV A ON1 *N* = 22	Other HRSV genotypes *N* = 10	*P* ^*∗*^
Demographic characteristics	*N* (%)^#^	*N* (%)^#^	*N* (%)^#^	

Gender				
M	21 (65.6)	16 (72.7)	05 (50.0)	0.21
F	11 (34.4)	06 (27.3)	05 (50.0)	

	Mean (sd)	Mean (sd)	Mean (sd)	

Age in months	2.45 (1.44)	2.33 (1.42)	2,71 (1,57)	0,51^##^

Clinical characteristics	*N* (%)^#^	*N* (%)^#^	*N* (%)^#^	

Cough	31 (100)	21 (100)	10 (100)	nsa
Fever	12 (37,5)	08 (38.1)	04 (40.0)	1.00^*∗∗*^
Dyspnea	23 (85.2)	17 (94.4)	06 (66.7)	0.09^*∗∗*^
Apnea	02 (06.3)	01 (9.09)	01 (25.0)	0.45^*∗∗*^
Complete Immunization for age^###^	24 (96.0)	18 (100)	06 (85.1)	0.28^*∗∗*^
Current Breastfeeding	26 (89.6)	18 (94.7)	8 (80.0)	0.27^*∗∗*^
Second-hand smoking	06 (18.8)	05 (33.3)	01 (14.3)	0.62^*∗∗*^
Admission in Critical Care Unit	05 (15.6)	03 (15.8)	02 (20.0)	0.64^*∗∗*^
Oxygen therapy	27 (87,1)	20 (95.2)	07 (70.0)	0.09^*∗∗*^
Mechanic ventilation	02 (6,45)	02 (09.1)	00 (00.0)	1.00^*∗∗*^

	Mean (sd)	Mean (sd)	Mean (sd)	

Duration of stay	5.13 (4.38)	5.91 (4.84)	3.40 (2.59)	0,14^##^

^#^Percentages are relative to the number of cases with complete information; ^*∗*^comparison of ON1 × other genotypes; ^*∗∗*^Fisher exact test; ^##^Student's *t*-test; ^###^according to Brazilian National Program of Immunization.
